# The Training and Work of a Hospital Almoner

**Published:** 1912-05-18

**Authors:** Edgar's Kemp

**Affiliations:** Secretary of the Hospital Almoners' Council.


### HOSPITAL OFFICERS: THEIR TRAINING AND WORK.

### The Training and Work of a Hospital Almoner.

By EDGAR S. KEMP, Secretary of the Hospital Almoners' Council.

"THE day is past when a hospital or any other charity may be regarded as a unit. Charity must now be regarded as a chain. The different forms of charity are links in that chain."

These words of Mr. E. W. Morris, Secretary of the London Hospital, in a paper on the work of hospital almoners, state briefly the claim that the work of the almoner makes for recognition by the hospital world. The almoner's work is to join together the links of charity, so that a combination of effort may secure a result otherwise unobtainable.

### THE WORK OF THE ALMONER.

It would be well, perhaps, to describe briefly the work of the almoner before referring to the training required: the necessity of a particular training will then be apparent. It is the business of the almoner to interview the hospital out-patients after they have been seen by a member of the staff. She will ensure, so far as possible, that they shall benefit to the full by the treatment they receive. With the co-operation of the patients, efforts will be made to secure the assistance of outside charitable agencies to provide the convalescent treatment, the surgical instruments, or the extra nourishment required. Sometimes advice only will be wanted—advice, for instance, as to the disadvantages of damp basements, of hermetically sealed windows, and of unsuitable food. Thus the doctor can understand the patient's home conditions, and the patient can be assisted to carry out the advice that the almoner's information has enbled the doctor to give. It is the almoner's business also to check the abuse of the out-patient department by those in a position to pay for treatment or by those too poor to benefit by any other than the complete provision made by the Poor Law. Any inquiries necessitated will be made through the agencies outside the hospital with which the almoner secures friendly co-operation. In return, she will undertake to obtain information for the clergy or others interested about the medical condition of patients attending the hospital. In this way she will save the hard-worked doctors much correspondence and trouble. She will be there to see that the patient, who is sent up by his own doctor, is attended to, and that the opinion of the hospital physician will reach the right quarter: thus she will extend the use of the out-patient department as a consulting centre. Finally, her office will act as a bureau of information on the innumerable questions which will arise from her work. It will be her business to know how people earning good wages can provide for their own medical treatment: she must be acquainted with the local friendly societies and their branches. The local apprenticeship committees, maternity clubs, relief societies, charity organisation committees, country holiday funds, invalid children's societies, military charities, hospital funds—all these must be known to her. She must understand their methods of working and the terms on which they are prepared to assist.

### THE TRAINING OF AN ALMONER.

This brief description of an almoner's duties should be sufficient to show that a particular kind of woman is needed to do this work, and that a particular kind of training is required to fit her for it. No one can become acquainted, by the light of nature, with a mass of technical detail. The Hospital Almoners' Council, which has for several years past been engaged in training women for almoner's posts, has adopted the following scheme of training. After being accepted for training, the candidate is sent for a minimum of six months to different district offices of the Charity Organisation Society. Here she is brought daily into contact with all the disabilities of fortune, health, and character, which go to make up the difficult corners in the lives of the poor; she learns at the same time what are the societies, the charities, the public authorities and the individuals who must be found to help, and what each will do. She is taught how to write letters, how to visit people in their homes, how to interview employers and others, and, finally, and most important, how to talk to those in difficulties, and how, sympathetically, to find the root of their troubles. This is the practical side of her work: the theoretical side will be taught her at the School of Sociology, where she will learn how to avoid missing the wood when she sees so many trees. After six months of this work, and if her progress is satisfactory—her work is regularly reported upon—she will be transferred to an almoner's office. Here she will learn all the problems that an almoner has daily to confront; she will assist in dealing with the stream of correspondence which flows in and out of an almoner's office. She will begin to understand the routine of a hospital; she will find how the medical staff is to be dealt with, what help will be expected from her, and what information she can obtain to offer. After six months' work of this kind, the Council may be prepared to recommend her for a post. Frequently she will obtain an assistant's post to begin with, and become an almoner later.

### The Selection of Candidates.

If training is necessary, it is clear also that the selection of a suitable candidate is an important matter. An almoner's work requires a level-headed woman, not liable to be flustered in emergencies, sympathetic but not weak, and, above all, tactful. If she is to be fully successful, she will need the consideration and support not only of the hospital governing body, of the secretary and of the medical staff, but also of the innumerable agencies and persons outside the hospital, on whom her work so much depends. If she is to elicit that support, she must show herself worthy of it, and must possess the necessary qualities which will secure it. Experience shows that women of good education, university or otherwise, with some experience of life, but without much to unlearn, with a real interest in social difficulties, and a capacity for establishing friendly relations with all kinds of people, make the best almoners. A sense of humour is also a valuable commodity. The Committee of the Almoners' Council interviews each candidate after she has been to a hospital for a few days to see something of what the work means, and endeavours to maintain a high standard among those selected for training.

### What the Almoner Means to a Hospital.

There are a number of considerations which occur to hospital boards when thinking of appointing an almoner. The writer has often been asked, "Will not she turn all our out-patients away? We do not want that." Or alternatively, "How much is she going to save us by getting rid of patients who should not come?" In answer to the first question, she will not turn all the out-patients away, and will turn no patient away without the doctor's consent. In answer to the second, little money will be saved owing to her dismissal of patients. The extent of the reduction of the number of out-patients must depend on their suitability, on social grounds, for treatment. The question of salary will naturally also be a matter for consideration.

### Men Almoners.

It will have been noticed that throughout this paper men almoners have not been referred to, and this for the reason that nearly all hospital almoners hitherto appointed have been women. It is work that women can undertake successfully, and it is possible to obtain for a commencing salary of £120 a year a much better class of woman than of man. Those who favour equal wages for equal work may object, but it is undeniable that this is the fact. As to the amount which should be at the disposal of the almoner for assisting patients, it has been pointed out that the great bulk of the monetary assistance required is obtained from the patients and their relatives and from charities outside the hospital. In a recently issued almoner's report it was shown that only one-seventh of the cost of providing surgical instruments came from the Samaritan Fund. "How many patients can an almoner see daily?" is a question frequently asked. The only reply can be that it depends upon the patients. Some will occupy much time, others little; but it may be added that the extent to which the almoner checks abuse cannot be estimated by the number of patients she dismisses. The mere fact that she is there will deter many who should not attend from coming at all.

The establishment of an almoner cannot be advocated on the ground that she will save the hospital money. That she may win the hospital increased support, especially if the work she does is brought prominently to the notice of its subscribers, there can be no doubt. But this, after all, is not the main point. A hospital cannot refuse to take advantage of the discoveries of medical science on the ground of expense: similiarly it will not be able to refuse to adopt a reform, which is, in its way, no less important. The question for hospital boards will be not "Can we afford an almoner?" but "Can we afford to do without one?"

